# Epidemiological, Clinical, and Laboratory Findings of 235 Hospitalized COVID-19 Adult Patients (Survivors and Non-Survivors) at Sohar Hospital in Oman

**DOI:** 10.7759/cureus.49157

**Published:** 2023-11-21

**Authors:** Awf Al Khan, Noora Al Balushi, Salima Al Maqbali, Elham Al Risi, Talib Al Maktoumi, Salman Al Mamari, Ayoob Al Balushi

**Affiliations:** 1 Department of Pathology and Blood Bank, Sohar Hospital, Sohar, OMN; 2 Department of Medical Statistics, Sohar Hospital, Sohar, OMN

**Keywords:** high ferritin, high troponin t, elevated c-reactive protein (crp), d-dimer, epidemiology, laboratory investigation, prognosis, survival, covid-19, coronavirus

## Abstract

Objectives: The aim of this study was to describe the epidemiological and clinical characteristics and laboratory findings of coronavirus disease 2019 (COVID-19) patients at the Sohar Hospital, Sohar, Oman.

Methods: This retrospective study of admitted COVID-19 patients at Sohar Hospital in Oman was carried out from March to October 2020. Demographics and laboratory data of 19 tests for 235 COVID-19 patients, of which 202 were survivors and 33 were non-survivors, were collected from the hospital information system after ethics approval.

Results: Thirteen factors were significantly correlated with in-hospital mortality, including older age, having chronic disease, high neutrophil count, high troponin T, high creatinine, low albumin (p < 0.0001), high white blood cell (WBC) count, low hemoglobin, high D-dimer (p < 0.001), high C-reactive protein (CRP) (p < 0.002), low lymphocyte count (p < 0.003), high alkaline phosphatase (ALP) enzyme (p < 0.007) and high ferritin (p < 0.045). The most common laboratory blood test abnormalities that were highly correlated with mortality were increased values of CRP (100% of non-survivors), D-dimer (94.1% of non-survivors), ferritin (88.2% of non-survivors), and troponin T (85% of non-survivors) and reduced lymphocyte count (73.9% of non-survivors).

Conclusion: These findings could help in categorizing COVID-19 patients for risk-based assessment and early identification of patients with poor prognosis.

## Introduction

The novel strain of coronavirus, called severe acute respiratory syndrome coronavirus- 2 (SARS-CoV 2), first emerged in Wuhan, China, in late 2019 [1]. After around one month, the new virus was transmitted to 19 different countries [1]. Human-to-human transmission outside of China was first recorded at the end of January 2020. World Health Organization (WHO) declared a public health emergency of global concern due to the rapid spread of coronavirus disease 2019 (COVID-19). After less than two months (March 11, 2020), as a result of the spread of COVID-19 dramatically increasing worldwide, WHO acknowledged that COVID-19 is a pandemic [2,3]. In Oman, the first case of COVID-19 was recorded in February 2020 and the first death was confirmed in March 2020 [4]. Until October 11, 2022, this new virus infected 627,018,121 patients and killed 6,562,012 worldwide [5].

COVID-19 has different clinical presentations, ranging from asymptomatic, mild, or severe symptoms to death [6]. The early identification of COVID-19 patients who are at high risk for poor outcomes is crucial for better management [6]. Therefore, it is vital to identify risk factors for early prediction of the severity of COVID-19 patients. 

Several studies stated that blood biomarkers could play a significant role in the diagnosis, staging, and treatment strategies of patients with COVID-19 [7,8]. Generally, it has been found that patients with a viral infection have low neutrophil count, low to normal concentration of C-reactive protein (CRP), and elevated concentration of myxoma resistance protein (MxA1) compared to patients with bacterial pneumonia [7]. A recent study revealed that levels of ferritin, procalcitonin (PCT), and CRP in serum were correlated with the severity of the COVID-19 infection [8]. In Chinese patients, a study showed that COVID-19 patients had increased concentrations of interleukin (IL) 1B, interferon (IFN) γ, interferon gamma-induced protein 10 (IP10), and monocyte chemoattractant protein 1 (MCP1) [2]. It has been also shown that age, serum lactate dehydrogenase (LDH), CRP, red blood cell distribution width (RDW), blood urea nitrogen (BUN), albumin, and direct bilirubin can be used as accurate prognostic indicators for COVID-19 patients [9].

A recent meta-analysis mentioned that studying alternation in values of biochemical and hematological tests can help in prognosis prediction of COVID-19 patients [10]. In line with these findings, several laboratory investigations have been identified as useful prognostic predictors and could help clinicians make a decision regarding the management of this disease [11,12]. Another method is to use a panel of blood biomarkers combined with clinical symptoms [7]. According to a study conducted by Huang et al., the most common symptoms of COVID-19 infection in Chinese patients were fever (98% of patients), cough (76% of patients), and myalgia or fatigue (44% of patients) [2]. Other less common symptoms were sputum production (28% of patients), headache (8% of patients), hemoptysis (5% of patients), and diarrhea (3% of patients) [2].

Blood biomarkers of COVID-19 and the link with in-hospital mortality have not yet been investigated amongst patients in the northern regions of Oman. Al Harthi et al. carried out a study at the central of Oman (Al Nahdha Hospital) to understand the sociodemographic, clinical, radiological, and laboratory features of the confirmed cases of COVID-19 comparing patients who required critical care and those who did not [13]. In order to further improve our understanding of the disease, this research has been conducted at Sohar Hospital, Sohar, Oman, with the aim to identify significant laboratory investigations that could play a role in early severity prediction of COVID-19 and the possibility to correlate with mortality.

## Materials and methods

Study design

This study is a retrospective study of admitted COVID-19 patients at Sohar Hospital in Sohar, Oman, between March 1 and October 30, 2020. The data was provided by the information technology department of Sohar Hospital via Al Shifa 3+ data system designed by the Ministry of Health in Oman. The study was approved by the Ministry of Health, Muscat, Oman (approval number: Moh/DGPS/CSR/PROPOSAL_APPROVED/39/2020).

Study population

All adult COVID-19 patients (>14 years old) admitted at Sohar Hospital between March 1 2020 and October 30 2020 were eligible to be included in this study. COVID-19 was confirmed by positive results of real-time reverse transcriptase-polymerase chain reaction (RT-PCR) (BD MAX™, Becton, Dickinson and Company, Franklin Lakes, New Jersey, United States) and Gene-Xpert (Cepheid, Sunnyvale, California, United States). Exclusion criteria were pregnant women, post-partum women, hospital staff, patients younger than 14 years old, patients discharged within 24 hours, and patients with less than two blood tests performed.

Data collection

The patient’s data was generated from the patient’s electronic medical records. It included demographic information such as age and gender, clinical presentation, and laboratory results. The clinical information consisted of the patient’s presenting symptoms such as fever, cough, myalgia or fatigue, sputum production, headache, diarrhea, difficulty in breathing, sore throat, and running nose, in addition to other information such as the presence of chronic illness, COVID-19 infection severity, and outcome (improved or died). The following lab investigation results were added to the study: full blood count (WBC count, neutrophil count, lymphocyte count, RDW, hemoglobin, and platelet count), D-dimer, CRP, renal function profile (serum sodium, serum potassium, serum chloride, serum carbon dioxide (CO2) and serum creatinine), liver function tests (serum total bilirubin, serum albumin, ALT, and ALP), ferritin and troponin T. Similar analyzers were used to study each group of the blood tests in the laboratory. Cobas® 8000, C502, and E602 (F. Hoffmann-La Roche AG, Basel, Switzerland) were used for biochemical tests, Alinity hq (Abbott Laboratories, Chicago, Illinois, United States) was used for hematological tests and Sysmex CS 2500 (Siemens Healthcare GmbH, Erlangen, Germany) was used for coagulation tests.

Statistical analysis

Data were analyzed using IBM SPSS Statistics for Windows, Version 24.0 (Released 2016; IBM Corp., Armonk, New York, United States). Data was summarized as frequencies and numbers for categorical variables or medians with interquartile ranges (IQRs) for continuous variables. Groups were compared using Mann-Whitney tests for continuous variables and the Chi-squared test for categorical data using a 95% confidence interval (CI) and p < 0.05 was considered statistically significant.

## Results

Epidemiological and clinical characteristics

Characteristics of hospitalized COVID-19 patients (survivors and non-survivors) are summarized in Table 1. Data were collected from 235 COVID-19 patients admitted to Sohar Hospital in the study period. Their ages ranged from 16 to 91 years with an average of 53 years. Of those, 81% were Omanis, male and female distribution was 55% and 45%, respectively, and 47.2% had no chronic diseases. The mortality rate was 14% and this was significantly higher in older COVID-19 patients compared to younger ones (p < 0.0001). It has been noticed that the mortality rate increased with age, reaching 72.7% for patients over 50 years old. There was a significant association between the mortality rate and having a chronic disease. The percentage of non-survivors with a chronic disease was 87.9%, which is higher than non-survivors with no chronic disease (12.1%). Similarly, the percentage of non-survivors with chronic diseases such diabetes, hypertension, renal disease, cardiovascular disease or chronic obstructive pulmonary disease was more than double the percentage of survivors who had chronic diseases (40.6%).

**Table 1:** Epidemiological data of 235 COVID-19 patients grouped according to patient outcome during the first seven months of the COVID-19 pandemic at Sohar Hospital, Oman Data presented as n(%); p < 0.05 was considered statistically significant. *Diabetes, hypertension, renal disease, cardiovascular disease or chronic obstructive pulmonary disease COVID-19: coronavirus disease 2019

Variables	Total population, n= 235	Survivors, n= 202 (86%)	Non-survivors, n= 33 (14%)	P-Value
Age (years old)	Number (%)	Number (%)	Number (%)	<0.0001
<30	20 (8.5%)	20 (9.9%)	0 (0%)	
30-39	59 (25.1%)	57 (28.2%)	2 (6.1%)	
40-49	52 (22.1%)	50 (24.8%)	2 (6.1%)	
50-59	31 (13.2%)	26 (12.9%)	5 (15.2%)	
60-69	38 (16.2%)	28 (13.9%)	10 (30.3%)	
70-79	25 (10.6%)	19 (9.4%)	6 (18.2%)	
>80	10 (4.3%)	2 (1.0%)	8 (24.2%)	
Gender				0.969
Male	131 (55.7%)	110(54.5%)	21 (63.6%)	
Female	104 (44.3%)	92 (45.5%)	12 (36.4%)	
Chronic disease*				<0.0001
Yes	111 (47.2%)	82 (40.6%)	29 (87.9%)	
No	124 (52.8%)	120 (59.4%)	4 (12.1%)	
Nationality				0.156
Omani	193 (82.1%)	163 (80.7%)	30 (90.9%)	
Non-Omani	42 (17.9%)	39 (19.3%)	3 (9.1%)	

The most common symptoms in COVID-19 survivors and non-survivors were fever (72.3%, 75.8%), cough (72.3%, 63.6%), and myalgia (37.1%, 39.4%), respectively (Table 2). Difficulty in breathing was a less common symptom in both groups. Other rare symptoms were headache, runny nose, sputum production, sore throat, and diarrhea. Table 2 shows that no significant difference was identified between the symptoms of survivors and non-survivors.

**Table 2:** Symptoms of COVID-19 survivors and non-survivors P < 0.05 was considered statistically significant COVID-19: coronavirus disease 2019

Symptoms	COVID-19 survivors , n (%)	COVID-19 non-survivors, n (%)	p-value
Headache	11 (5.4%)	1 (0.3%)	0.559
Runny nose	8 (4.0%)	0 (0%)	0.245
Sputum production	15 (7.4%)	0 (0%)	0.106
Cough	146 (72.3%)	21 (63.3%)	0.310
Sore throat	20 (9.9%)	4 (12.1%)	0.696
Difficulty in breathing	50 (24.8%)	9 (27.3%)	0.757
Fever	146 (72.3%)	25 (75.8%)	0.677
Myalgia	75 (37.1%)	13 (39.4%)	0.803
Diarrhea	11 (5.4%)	0 (0%)	0.151

Laboratory findings

Results of the laboratory tests for COVID-19 survivors and non-survivors admitted to Sohar Hospital in the study period are presented in Table 3. The most common abnormal laboratory results in both COVID-19 survivors and non-survivors were increased values of CRP (87%), ferritin (69.9%), and D-dimer (63.8%), and reduced levels of serum CO2 (50%) and lymphocyte count (49%).

**Table 3:** Laboratory investigations for admitted COVID-19 patients (survivors and non-survivors) In non-survival patients, WBC count, neutrophil count, D-dimer, CRP, ferritin, troponin T, ALP, and creatinine levels were significantly higher compared with survival COVID-19 patients. On the other hand, lymphocyte count, hemoglobin, and albumin levels were significantly lower. P < 0.05 was considered statistically significant. WBC: white blood cells; RDW: red cell distribution width; PLT: platelets; CRP: C-reactive protein; ALT: alanine transaminase; ALP: alkaline phosphatase; COVID-19: coronavirus disease 2019

Variables	Total population (n= 235)	Survivors, n= 202 (86%)	Non-survivors, n= 33 (14%)	p-value
	Number (%)	Number (%)	Number (%)	
WBC count >10 x 10^3^/uL	18 (19.8%)	9 (12%)	9 (56.3%)	0.001
Neutrophil count >5 x 10^3^/uL	90 (38.5%)	69 (34.2%)	21 (65.6%)	<0.0001
Lymphocyte count <1.2 x 10^3^/uL	70 (49.0%)	53 (44.2%)	17 (73.9%)	0.003
RDW <11.5% >16.5%	10 (7.0%) 10 (7.0%)	9 (7.1%) 8 (6.3%)	1 (6.3%) 2 (12.5%)	0.663
Hemoglobin <11.5 g/dl	48 (33.6%)	33 (27.5%)	15 (65.2%)	0.001
PLT count <140 x 10^3^/uL	18 (12.6%)	13 (10.8%)	5 (21.7%)	0.733
D-dimer >0.5 mg/l	60 (63.8%)	44 (57.1%)	16 (94.1%)	0.001
CRP >5 mg/l	188 (87.0%)	156 (84.8%)	32 (100%)	0.002
Ferritin >400 ug/l	51 (69.9%)	36 (64.3%)	15 (88.2%)	0.045
Troponin T >14 pg/ml	34 (31.2%)	17 (19.1%)	17 (85.0%)	<0.000
Sodium <136 mmol/l	53 (37.9%)	45 (36.3%)	8 (50.0%)	0.387
Potassium >5.1 mmol/l	17 (12.8%)	14 (11.9%)	3 (20.0%)	0.289
Chloride <97 mmol/l	48 (34.3%)	40 (32.3%)	8 (50.0%)	0.284
CO_2_ in serum <22 umol/l	70 (50%)	60 (48.4%)	10 (62.5%)	0.287
Creatinine >106 umol/l	39 (17.1%)	21 (10.7%)	18 (56.3%)	<0.0001
Total bilirubin >20 umol/l	14 (6.6%)	7 (3.9%)	7 (22.6%)	0.104
Albumin <35 g/l	57 (26.6%)	37 (20.3%)	20 (66.7%)	<0.0001
ALT >41 U/l	74 (35.2%)	61 (34.3%)	13 (40.6%)	0.729
ALP >129 U/l	14 (10.6%)	10 (9.2%)	4 (17.4%)	0.007

**Figure 1:**
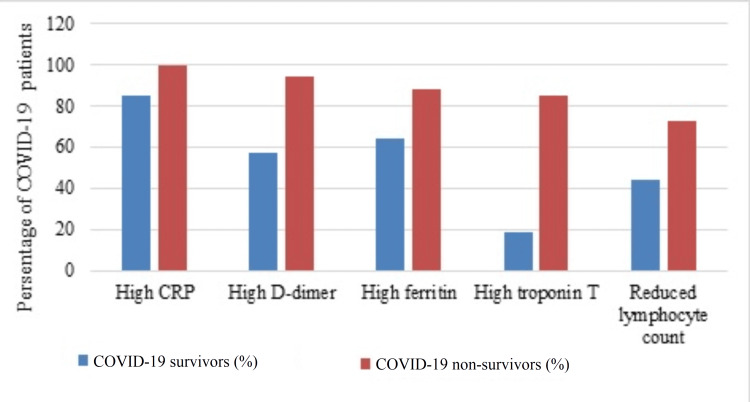
The most common laboratory blood test alterations in COVID-19 non-survivors compared to survivors. COVID-19: coronavirus disease 2019; CRP: C-reactive protein

Significant differences in some laboratory blood tests between the COVID-19 survivors and non-survivors are illustrated in Table 3. In non-survivors, WBC count (p = 0.001), neutrophil count (p < 0.0001), D-dimer (p = 0.001), CRP (p = 0.002), ferritin (p = 0.045), troponin T (p < 0.0001), ALP (p = 0.007), and creatinine levels (p < 0.0001) were significantly higher compared to survivors. On the other hand, lymphocyte count (p = 0.003), hemoglobin (p = 0.001), and albumin levels (p < 0.0001) were significantly lower. The most common laboratory alterations that highly correlated with mortality were increased values of CRP (100%), D-dimer (94.1%), ferritin (88.2%), and troponin T (85%), and reduced lymphocyte count (73.9%) (Figure 1). No significant correlations were observed for RDW, platelet count, sodium, potassium, chloride, CO2 in serum, total bilirubin, and ALT levels.

## Discussion

The results of laboratory blood tests can be used with clinical data to predict the severity of COVID-19 and could improve prognosis via better management of the disease. Nevertheless, more research is required to better elucidate these alternations in the laboratory tests and their links with the prognosis [14]. In this study, characteristics and factors that could be related to in-hospital mortality among 235 COVID-19 patients at Sohar Hospital were described focusing on the alternations in 19 blood tests. The study found that factors associated with in-hospital mortality were older age, having chronic disease, high neutrophil count, high troponin T, high creatinine, low albumin, high WBC count, low hemoglobin, high D-dimer, high CRP, low lymphocyte count, high ALP, and high ferritin. The most common laboratory blood test abnormalities that were linked with mortality were increased values of CRP, D-dimer, ferritin, and troponin T and reduced lymphocyte count.

In line with another study [6], the current study found that the rate of in-hospital mortality was 14%, while other national [15] and international studies reported that the mortality rate ranged from 11.9% to 28% [16,17]. These differences are also seen in other international studies of hospital-admitted COVID-19 patients. For example, in the United Kingdom, the mortality rate was 26% [18] and in China it ranged from 1.4% [19] to 17% [20]. These variations in the rate of mortality could be due to the differences in the presence of comorbidities, demographic properties, methods used for the diagnosis, and ways of identification of COVID-19-linked death [21]. Other causes could be related to variations in the inclusion criteria of patients in each study, health resources availability, and criteria of hospital admission [6].

The mortality rate was the highest among patients over 50 years old, which is similar to studies conducted in China [19] and in Oman [13,22]. A British study [18] and Spanish studies [6,16,17] showed the highest mortality in older age groups ranging from 66 to 73 years. Similar to the current study, international [6,23] and national [15,22] studies found that the mortality rate is higher in older patients compared to younger patients. These variations could be related to the differences in average life span in each country.

Contrary to this study, it has been noted that sex was significantly correlated to poor prognosis of COVID-19; thus, the rate of mortality was higher in males compared to females [16-18]. In the current study, the insignificant difference within the sex group could be related to the small sample number.

Having one or more chronic diseases was described as a risk factor for the severity of COVID-19 [24]. Similar to previous international studies [16-18], our study showed that the rate of mortality was high for patients with comorbidity, with around 88% of non-survivors having one or more chronic diseases including diabetes, hypertension, renal disease, cardiovascular disease, or chronic obstructive pulmonary disease. Unfortunately, the correlation between each single chronic disease and mortality rate was not studied in this research. Data related to obesity or dyslipidemia was also not collected in this study.

Fever and cough were the most common symptoms in COVID-19 patients. Other uncommon symptoms were myalgia, difficulty in breathing, and sore throat. These findings agree with previous national [13,15,22,25] and an international study [2].

This study showed that the most common alterations were increased values of CRP, ferritin, D-dimer, reduced amount of CO2 in serum, and reduced lymphocyte count, and these could be considered as significant biomarkers for COVID-19 prognosis. These findings agree with recent national [15] and international studies [6,26]. Similar to our findings, Wang et al. reported that increased values of CRP, D-dimer, ferritin, and troponin T and reduced lymphocyte count were the most frequent laboratory alterations that were highly associated with mortality [27]. Alterations in these inflammatory biomarkers could be linked with the cytokine storm which has already been identified in COVID-19 patients with poor prognosis [27]. Furthermore, a significant number of patients show abnormal values of other blood tests even in the early stages of the disease [27]. These alterations were seen with the following parameters: WBC, neutrophil, ALP, creatinine, hemoglobin, and albumin [6,17] Unfortunately, the current study could not investigate the blood levels of procalcitonin and lactate dehydrogenase (LDH), which were found to be good biomarkers and involved in the prognosis of COVID-19 [6,15,17,18]. This suggests that these two markers may play a role in determining the outcome of the disease. Similar to the current study, Bhargava et al. investigated the role of creatinine as a significant biomarker for poor prognosis of COVID-19 [28]. They reported that acute renal disease is a predictor of poor outcomes of COVID-19 [28]. Another study attempted to explain the mechanism of acute renal injury in patients with COVID-19 [29]. They suggested that the virus uses angiotensin-converting enzyme 2 (ACE2) receptor to enter renal cells and cytokines that are induced as a response for this virus cause indirect effects on these cells through hypoxia or shock [29].

To our knowledge, this is the first study that compared the results of laboratory blood tests between a reasonable number of COVID-19 survivors and non-survivors in the northern region of Oman. Using of single analyzer for each group of blood tests ensured results comparability. Nevertheless, the current study has some limitations, for instance, possible errors in clinical data entry, sample selection, and inclusion criteria. Requests for blood tests varied from one doctor to another. Furthermore, a shortage of some reagents resulted in getting incomplete test profiles for some patients and the number of cases tested for a specific laboratory test was not the same. In addition, procalcitonin, IL-6, IL1B, INF γ, IP10, and MCP1 serum, and LDH, which were reported earlier as significant predictors for the severity of COVID-19 [2,8,9,15,30] were not investigated in the current study. Most of these tests are not carried out at the laboratory of Sohar Hospital except LDH, which was tested in a minor number of patients and excluded from the data analysis.

## Conclusions

This study identified significant predictors for COVID-19 severity, including older age, presence of chronic disease, high WBC count, high neutrophil count, low lymphocyte count, low hemoglobin, high D-dimer, high CRP, high ferritin, high troponin T, high creatinine, low albumin, and high ALP. Besides, this study showed that the most common laboratory blood test alternations that were strongly associated with in-hospital mortality are increased levels of CRP, D-dimer, ferritin, and troponin T and reduced lymphocyte count. These findings suggested that these blood biomarkers could help in categorizing COVID-19 patients for risk-based assessment, early identification of patients at a higher risk for poor prognosis, better patient management, and improved admission criteria.
